# Genomic population structure and local adaptation of the wild strawberry *Fragaria nilgerrensis*

**DOI:** 10.1093/hr/uhab059

**Published:** 2022-01-19

**Authors:** Yuxi Hu, Chao Feng, Lihua Yang, Patrick P Edger, Ming Kang

**Affiliations:** 1 Key Laboratory of Plant Resources Conservation and Sustainable Utilization, South China Botanical Garden, Chinese Academy of Sciences, Guangzhou 510650, China; 2 University of Chinese Academy of Sciences, Beijing 100049, China; 3 Department of Horticulture, Michigan State University, East Lansing, MI 48824, USA; 4 Center of Conservation Biology, Core Botanical Gardens, Chinese Academy of Sciences, Guangzhou 510650, China

## Abstract

The crop wild relative *Fragaria nilgerrensis* is adapted to a variety of diverse habitats across its native range in China. Thus, discoveries made in this species could serve as a useful guide in the development of new superior strawberry cultivars that are resilient to new or variable environments. However, the genetic diversity and genetic architecture of traits in this species underlying important adaptive traits remain poorly understood. Here, we used whole-genome resequencing data from 193 *F. nilgerrensis* individuals spanning the distribution range in China to investigate the genetic diversity, population structure and genomic basis of local adaptation. We identified four genetic groups, with the western group located in Hengduan Mountains exhibiting the highest genetic diversity. Redundancy analysis suggested that both environment and geographic variables shaped a significant proportion of the genomic variation. Our analyses revealed that the environmental difference explains more of the observed genetic variation than geographic distance. This suggests that adaptation to distinct habitats, which present a unique combination of abiotic factors, likely drove genetic differentiation. Lastly, by implementing selective sweep scans and genome–environment association analysis throughout the genome, we identified the genetic variation associated with local adaptation and investigated the functions of putative candidate genes in *F. nilgerrensis.*

## Introduction

Crop wild relatives (CWRs), commonly defined as the progenitors and other close relatives of agricultural and horticultural crops, contain a reservoir of beneficial traits for crop improvement and food security [1–3]. The market demand for high productivity and uniformity have exacerbated the reduction of genetic diversity during crop domestication [3]; on the contrary, CWRs have not passed through the bottlenecks of domestication and have the ability to adapt to diverse environment conditions [4]. Over the past decades, a series of important traits, such as pest or disease resistance, abiotic stress tolerance, increased nutritional value, higher yield, or production stability, have been successfully introduced from CWRs into crops [1, 5]. However, due to climate change and increasing human activities, a significant proportion of CWR species are currently threatened with genetic erosion or extinction to varying degrees [6, 7]. Given the vital status of these species in broadening the genetic base of crops, it is critical to understand their genetic diversity and adaptability to their habitats.

**Figure 1:**
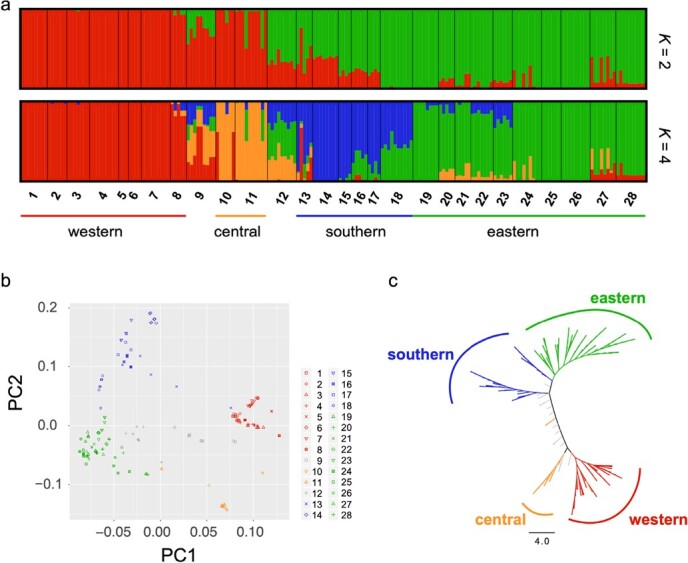
**a** Population structure of *F. nilgerrensis* identified with STRUCTURE based on genome-wide SNPs. *K* = 2 shows the highest ∆*K*, and *K* = 4 represents the fine-scale structure within *F. nilgerrensis*. **b** PCA for all populations based on the same SNP data set as STRUCTURE. (c) Neighbor-joining phylogenetic tree of all samples.

**Figure 2:**
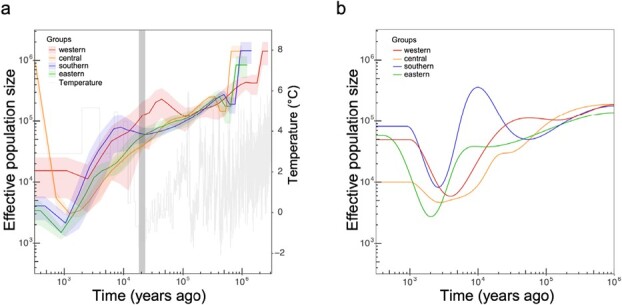
Historical changes in effective population size of the four *F. nilgerrensis* groups. **a** Inferred using MSMC based on sets of four haplotypes, with solid lines representing medians and shading representing ± standard deviation calculated across pairs of haplotypes. The dark gray bar indicates the period of the LGM. **b** Inferred using SMC++ based on individuals in each group

**Figure 3:**
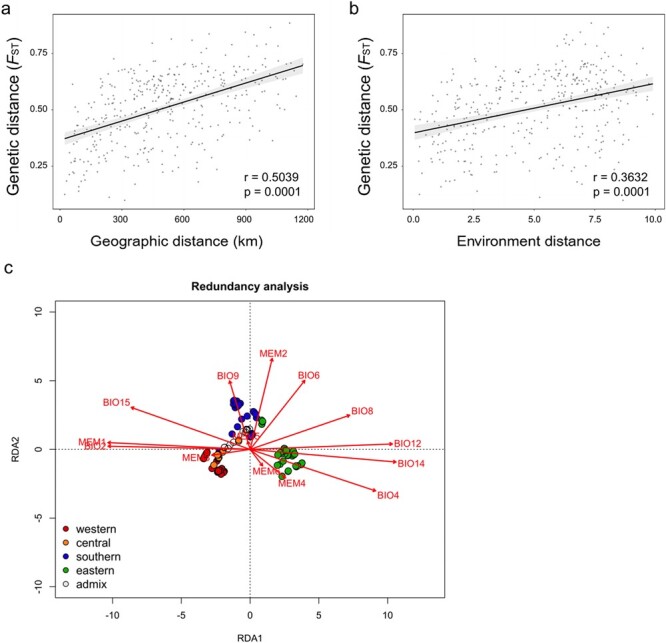
IBD, IBE, and RDA. **a** Genetic pairwise differentiation plotted against geographic distances. **b** Environmental distances between populations. **c** RDA testing the effect of geographic and environmental variables on the degree of genetic differentiation. The first two canonical axes (RDA1 and RDA2) are shown.

**Figure 4:**
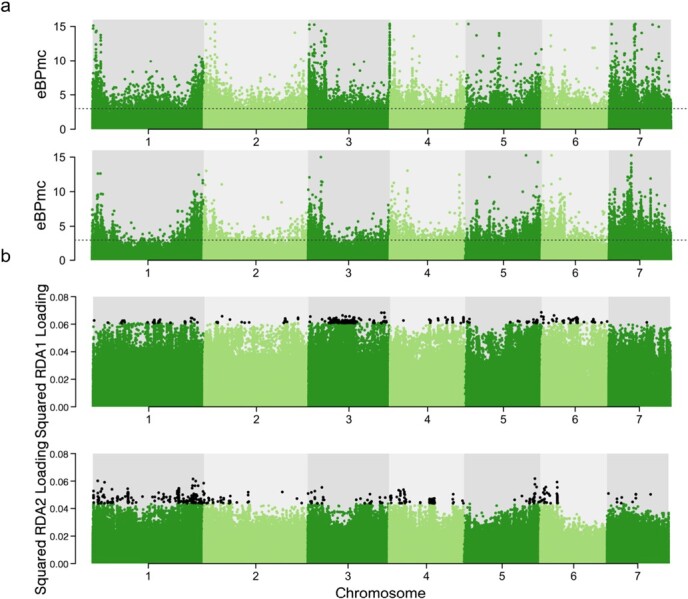
Manhattan plots for the results of GEA. **a** Empirical Bayesian *P*-value (eBPmc) for an association with the first environment principal component (top panel) and the second environment principal component (bottom panel). The dotted black line establishes the eBPmc significance threshold at 3. **b** SNP loadings on the first RDA axis (top panel) and the second RDA axis (bottom panel) accounting for spatial structure among populations. The black dots represent SNPs with significant associations along the RDA axes (at least three standard deviations away from the mean squared loadings). We only show the first two RDA axes here.

**Table 1:** Summary information on the sampling locations and genetic diversity estimates for the 28 populations of *F. nilgerrensis*.

**Region/population**	***N***	**Location**	**Latitude (°N)**	**Longitude (°E)**	**Altitude (m)**	**Nucleotide diversity (*π*)**	**Tajima’s *D***
Western						0.00567 ± 0.00076	−0.1199 ± 0.00431
1. YLH708	8	Yanbian, Sichuan	27.1396	101.3292		0.00190 ± 0.00057	1.2640 ± 0.00893
2. YLH813	6	Ninglang, Yunnan	27.5246	100.8037	2558	0.00264 ± 0.00046	1.0918 ± 0.00864
3. YLH811	7	Muli, Sichuan	27.6868	101.2223	3247	0.00483 ± 0.00127	−0.0342 ± 0.00941
4. YLH714	9	Yanyuan, Sichuan	27.7918	101.4183	3193	0.00374 ± 0.00177	0.7077 ± 0.01136
5. YLH701	3	Weixi, Yunnan	27.3238	99.2757	2861	0.00616 ± 0.00093	1.7290 ± 0.00451
6. YLH698	4	Deqin, Yunnan	28.0094	98.8796	2803	0.00057 ± 0.00010	0.7224 ± 0.01000
7. YLH696	9	Lushui, Yunnan	25.8388	99.1040	2574	0.00236 ± 0.00049	0.3221 ± 0.00903
8. YLH752	5	Jinyang, Sichuan	27.8139	103.1847	3081	0.00583 ± 0.00170	1.2166 ± 0.00601
Central						0.00317 ± 0.00070	−0.6539 ± 0.00776
10. YLH749	6	Leibo, Sichuan	28.3169	103.6261	1360	0.00425 ± 0.00132	0.1680 ± 0.00858
11. YLH760	10	Ludian, Yunnan	27.2976	103.3976	2312	0.00206 ± 0.00042	0.2040 ± 0.00952
Southern						0.00268 ± 0.00037	−1.2180 ± 0.00750
13. YLH768	5	Huize, Yunnan	26.2954	103.2274	3119	0.00481 ± 0.00084	−0.5972 ± 0.00664
14. YLH676	8	Jingdong, Yunnan	24.3735	100.7535	2060	0.00121 ± 0.00033	1.0975 ± 0.00968
15. YLH821	4	Yuxi, Yunnan	24.3088	102.3557	2072	0.00119 ± 0.00038	0.3656 ± 0.01055
16. YLH842	5	Yuanyang, Yunnan	23.0460	102.8980	2480	0.00184 ± 0.00017	1.1484 ± 0.00787
17. YLH850	4	Wenshan, Yunnan	23.5593	103.9429	2377	0.00172 ± 0.00021	0.1981 ± 0.01068
18. YLH773	10	Luoping, Yunnan	24.8760	104.2599	1927	0.00112 ± 0.00024	0.3121 ± 0.01527
Eastern						0.00205 ± 0.00058	−0.9563 ± 0.00891
19. YLH776	8	Anlong, Guizhou	25.3658	105.5136	1458	0.00092 ± 0.00021	0.7832 ± 0.01046
20. YLH602	5	Guiding, Guizhou	26.2086	107.0325	1225	0.00142 ± 0.00081	0.8316 ± 0.01039
21. YLH603	5	Dushan, Guizhou	25.9542	107.6286	1340	0.00093 ± 0.00081	0.4319 ± 0.01103
22. YLH599	7	Leishan, Guizhou	26.3789	108.1917	1785	0.00098 ± 0.00031	0.4686 ± 0.01103
23. YLH580	6	Xianfeng, Hubei	29.4153	108.9828	1295	0.00139 ± 0.00055	0.5281 ± 0.01259
24, YLH576	9	Qianjiang, Chongqin	29.6264	108.4767	1117	0.00140 ± 0.00052	−0.3732 ± 0.01101
25. YLH526	6	Hefeng, Hubei	30.0614	110.0675	1052	0.00060 ± 0.00034	0.8914 ± 0.01022
26. YLH585	9	Longshan, Hunan	28.8386	109.2514	1200	0.00072 ± 0.00037	0.8365 ± 0.01339
27. YLH528	8	Xuanen, Hubei	29.9772	109.7495	1813	0.00297 ± 0.00157	−0.3891 ± 0.01343
28. YLH533	9	Fengjie, Chongqin	30.5391	109.3560	1581	0.00163 ± 0.00129	−0.6183 ± 0.01620
Admixture							
9. YLH756	9	Qiaojia, Yunnan	27.0872	103.0043	2662	0.00436 ± 0.00086	0.3485 ± 0.00641
12. YLH762	9	Weining, Guizhou	26.7728	103.9721	2078	0.00308 ± 0.00049	0.6963 ± 0.00802

**Table 2:** Pairwise genetic differentiation (*F*_ST_) values between the four groups of *F. nilgerrensis* based on the sequence data.

**Group**	**Western**	**Central**	**Southern**
Central	0.29295 ± 0.00091		
Southern	0.30212 ± 0.00094	0.48538 ± 0.00161	
Eastern	0.39402 ± 0.00104	0.54958 ± 0.00166	0.25091 ± 0.00144

The cultivated garden strawberry (*Fragaria × ananassa*) is one of the most economically and commercially important fruits throughout the world. The Fruits are rich in a variety of nutritive compounds, including vitamin C, folate, minerals, and dietary fibers, and are a valuable source of phenolic compounds, which are known to have antioxidant and anti-inflammatory properties [8]. Therefore, the potential positive impact of strawberry consumption on human health and disease prevention remains an active research area [9]. However, cultivated strawberries have a short shelf life and limited hardiness resistance, and occupy a prominent position on the list of foods with the highest pesticide residues [10]. In addition, modern strawberry breeding has problems such as a narrow parental genetic background and a lack of phenotypic diversity present in most breeding programs. The genus *Fragaria* contains 16 priority CWRs for improving cultivated strawberry with the potential to improve fruit quality traits, abiotic stress tolerance, and biotic resistance [2, 11]. These wild relatives are naturally distributed across the northern hemisphere, with China being the crucial center of diversity [12, 13]. Among the CWRs of strawberry, *Fragaria nilgerrensis* is a self-compatible diploid species widely distributed in central and southwest China. The mature fruits of *F. nilgerrensis* are white to cream, with a somewhat banana-like taste and a fruity aroma [14, 15]. In addition, *F. nilgerrensis* possesses some desirable characteristics that can be used for cultivated strawberry breeding, such as leaf disease resistance and waterlogging resistance [15, 16]. For example, Noguchi [17] used *F. nilgerrensis* and *F. × ananassa* to obtain an interspecific decaploid hybrid, ‘Tokun’, with a unique blend of peach and coconut aromas. However, little is known about the patterns of genetic structure in *F. nilgerrensis* and the genetic basis of adaptive differences among populations within this species.

With the advent of cost-effective next-generation sequencing technologies, a growing quantity of genome-wide data is becoming available, especially for non-model organisms. Such methodological progress has allowed the improved characterization of genetic variation and population structure at a genome-wide level [18, 19]. Whole-genome resequencing approaches are increasingly applied to investigate the genome variation and population structure of various plant species, including *Populus davidiana* [20], cushion willow [21], and ginkgo trees [22]. Furthermore, it is now possible to study a vast number of loci providing unprecedented insights into the genome-wide effects of accumulating genetic divergence and the molecular basis of adaptation [23–27]. Previous studies have shown that adaptation to local environments has contributed to the observed phenotypic variation within species, which are distributed over heterogeneous environments across their geographic range [28, 29]. Nevertheless, the mechanisms through which organisms adapt to heterogeneous natural environments remain poorly understood [30]. The reliability and power of whole-genome single-nucleotide polymorphism (SNP) data for investigating natural populations are well established, and SNP identification and genotyping have become a routine [31, 32]. Whole-genome resequencing data have been increasingly used to infer the genetic basis of adaptively important traits [33, 34] or to detect potential local adaptive genetic variants associated with environmental variables [35, 36]. However, the application of whole-genome sequencing analysis in CWR species is still limited. The recent release of the high-quality genome of *F. nilgerrensis* [37] provides a novel opportunity to investigate genomic variation and local adaptation of this species.

In this study, we focus on investigating the population structure and the genetic basis of local adaptation in *F. nilgerrensis* with whole-genome resequencing data generated from 193 samples from 28 populations spanning the distribution range of *F. nilgerrensis* in China. First, we characterized the genetic population structure and historical demographic process. Second, we aimed to identify the likely drivers of genetic divergence across its native range and evaluate the relative contribution of environmental and geographical factors to genetic variation. Finally, after implementing selective sweep scans and genome-environment association analysis, we identified regions across the genome containing putative candidate genes associated with local adaptation to certain ecological niches.

## Results

### Genome sequencing and SNP calling

We obtained whole-genome resequencing data for 193 samples from 28 populations of *F. nilgerrensis*, with average sequencing depth of ~43× per individual covering >96.87% of the reference genome (Table 1; Supplementary Table S1). After variant calling and subsequent stringent filtering, we obtained a total of 9 499 952 high-quality SNPs.

### Population structure and genetic diversity

We first performed clustering analysis using STRUCTURE to assess the population structure of *F. nilgerrensis*. Using the Δ*K* method, the highest Δ*K* value identified was *K* = 2 (Fig. 1a) and the second highest value was *K* = 4 (Supplementary Fig. S1), which exhibited a fine-scale structure within *F. nilgerrensis*. Given the high *F*_ST_ among populations (Supplementary Table S2) and the potential bias of the Δ*K* method [38], we focused our analysis on the four genetic groups that are defined herein as western, central, southern, and eastern groups (Fig. 1a). Because two populations (populations 9 and 12) showed a high degree of admixture (Table 1), we excluded them from the subsequent group-level local adaptation analysis. Principal component analysis (PCA) as well as a neighbor-joining (NJ) tree analysis further supported the four genetic groups (Fig. 1b and c) mirroring the geographical distribution pattern. The western group was distributed in the Hengduan Mountains, specifically in the north of Yunnan Province and the south of Sichuan Province. The central group was distributed to the east of the Hengduan Mountains, mainly in the northeast of Yunnan Province. The southern group was distributed in Yunnan Province, and the eastern group was relatively widely distributed, involving the four provinces of Guizhou, Chongqing, Hunan, and Hubei. All pairs of groups were substantially differentiated from one another, with pairwise *F*_ST_ values ranging from 0.25091 to 0.54958 (Table 2).

Within each genetic group, the western group exhibited the highest nucleotide diversity (*π* = 0.00567), whereas the eastern group had the lowest (*π* = 0.00205) despite the large geographic area covered by this group. Average Tajima’s *D* value was slightly negative in the western group (Tajima’s *D* = −0.1199), suggesting slight expansion or weak selection in this group. The remaining three groups had relatively strong negative Tajima’s *D* values (Table 1), which may imply that stronger selection or population expansion occurred.

### Demographic history

We evaluated the effective population size (*N*_e_) over historical time for the four groups. We first used a multiple sequentially Markovian coalescent method (MSMC) to infer the demographic history based on sets of four haploid genomes for each group. The results showed that all groups experienced a period of population decline since the inferred origin of each group (Fig. 2a). The western group experienced a population expansion since the last ice age (110 Kya), and the southern group experienced a population expansion after the last glacial maximum (LGM, 23–18 Kya), whereas the other two groups, central and eastern, continued to show clear signs of notable population contraction. These four groups showed a general pattern of population decline during the entire period but expanded recently (Fig. 2a). We then used the sequentially Markovian coalescent method in SMC++, which can provide more accurate estimates for relatively more recent historical events. Overall, the trends presented in the SMC++ results were consistent with the MSMC analysis (Fig. 2b). However, interpretation of the exact estimated value of *N*_e_ should be cautious, and the historical trend of the population should be considered instead of the exact value of each curve.

### Effects of geographic and climatic factors on genomic variation

To evaluate the effect of geographic and climatic factors on shaping genomic variation among populations, we tested isolation by distance (IBD) and isolation by environment (IBE). We identified a significant correlation between pairwise *F*_ST_ and geographic distance (*r* = .5039, *P* = .0001; Fig. 3a), which indicates a significant pattern of IBD. A significant pattern of IBE was also detected (*r* = 0.3632, *P* = .0001; Fig. 3b). Considering the strong autocorrelation between environmental and geographic distances (*r* = .7098, *P* = .0001; Supplementary Fig. S2), we further performed redundancy analysis (RDA) to assess the relative contributions of geographic and climatic factors driving genetic variation patterns (Fig. 3c).

After filtering and forward selection, we identified eight climate variables as significantly predictive of the standing genetic variation observed among populations. These climate variables included BIO2 (mean diurnal range), BIO4 (temperature seasonality), BIO6 (minimum temperature of coldest month), BIO8 (mean temperature of wettest quarter), BIO9 (mean temperature of driest quarter), BIO12 (annual precipitation), BIO14 (precipitation of driest month), and BIO15 (precipitation seasonality). Forward selection of the distance-based Moran’s eigenvector map (dbMEM) variables identified six axes as significant to explain geographical structure among populations. Retained dbMEM variables included dbMEM1, 2, 3, 4, 5, and 6, among which dbMEM1 contributed most of the variation (2.39%) (Supplementary Table S3) and represented a broad-scaled structure (Supplementary Fig. S3). The redundancy analysis revealed that the abiotic environment and geographic variables explained 52.12% of the genetic variation among populations (Supplementary Table S3), with 28.29% associated with the collinear portion of environment and geography. The variance partitioning test showed that the contribution of environmental variables to genetic variation was slightly higher than that of geographic variables (12.25 and 11.58% respectively; Supplementary Table S3).

### Selective sweeps

To search for genomic regions that have undergone recent positive selection, containing key adaptive genes, we performed genome scans using a composite evaluation method (RAiSD) and a haplotype-based method (XP-nSL) for each group separately. The overlapped regions between these two methods were considered as the candidate regions for subsequent analyses. We revealed a total of 344 genomic regions for all four groups (63, 81, 76, and 124 regions for the western, central, southern, and eastern groups, respectively; Supplementary Table S4). Using gene annotations from *F. nilgerrensis*, we identified a total of 959 genes that were located within selective sweeps, including 159, 245, 165, and 390 genes for the western, central, southern, and eastern groups, respectively. Of the 959 genes, a total of 859 (89.57%) were identified as under selection in single groups, while the remaining 100 genes were identified as shared by two groups, and no genes were shared by three or more groups (Supplementary Fig. S4). This suggests that unique adaptive patterns exist for each group, and that these gene differences arose to permit adaptation to unique climates.

To further investigate these candidate genes, we performed a gene ontology (GO) enrichment analysis using our *F. nilgerrensis* annotation. We found that genes related to response to external stimuli, wounding, and stresses were overrepresented in the western group. (Supplementary Table S5), suggesting the importance of these stress-related genes to adapt to highly heterogeneous alpine environments in the Hengduan Mountains. Several other GO terms related to cation/ion transmembrane transport, ion transport, and dephosphorylation were also notable as potentially resistance-related (Supplementary Table S5). In the central group, genes found in selective regions were particularly enriched for regulatory functions, such as positive regulation of RNA metabolic process, positive regulation of RNA biosynthetic process, and positive regulation of gene expression (Supplementary Table S5), which might be related to environmental adaption to the transitional areas between high and low altitudes. In the southern group, highly enriched GO categories were tryptophan metabolic/biosynthetic process, indole-containing compound metabolic/biosynthetic process, and actin filament bundle assembly/organization (Supplementary Table S5), which might be linked to regulating plant development and growth, pathogen defense responses, and plant–insect interactions. In the eastern group, GO terms related to arginine metabolic/biosynthetic process and cellular polysaccharide metabolic/biosynthetic process were highly enriched (Supplementary Table S5). These GO terms might be connected to growth, stress protection, and signal transduction. The enrichment of GO terms related to plant development and bacterial defense response in the southern and eastern groups might be associated with adaptation to the relatively low-altitude environment.

### Identification of genome–environment associations

To elucidate the pattern of adaptation, we further performed genome–environment association (GEA) analysis to narrow down the genomic regions containing selective sweeps. Specifically, we considered only overlaps between selective sweeps and those showing significant environmental associations. We identified SNPs associated with climatic variables using two GEA methods: the BayPass standard covariate model and partial RDA. BayPass uses a matrix of covariances of allele counts to account for underlying population structure. The 19 climatic variables showed a high degree of correlation (Supplementary Fig. S5a). After performing PCA on climatic variables, we retained the first two principal components for climatic association analysis (Supplementary Fig. S5b). The first environmental principal component explained 55.9% of the total variance (Supplementary Fig. S5b and c), and had the strongest loadings for the mean temperature of warmest quarter, followed by the precipitation of driest month, the maximum temperature of warmest month, the precipitation of driest quarter, and the annual precipitation (Supplementary Figs S5d and S6a). The second environmental principal component explained 28.8% of the total variance (Supplementary Fig. S5b and S6c), with the mean temperature of coldest quarter contributing the most, followed by the mean temperature of driest quarter (Supplementary Figs S5d and S6b). The BayPass STD model identified a total of 21 796 SNPs having significant correlations with the climatic variables (Fig. 4a). For our partial RDA with eight climatic variables, the conditional variance explained 43.61%, the constrained variance explained 14.45%, and the unconstrained variance explained 41.95% of the total variance. We identified candidate loci associated with local adaptation by inspecting SNPs displaying loadings along the first four RDA axes ± 3 SD from the mean, and a total of 23 263 SNPs were identified displaying strong associations with climatic variables (Fig. 4b; Supplementary Fig. S7).

In order to obtain a conservative list of genes under selection potentially related to adaptation to different climatic environments, we further focused on the genes that were found in the regions of selective sweeps and also showed significant climatic associations. The outlier SNPs detected by the BayPass STD model involved 3677 genes, and the outlier SNPs detected by partial RDA involved 3977 genes. From this analysis 186 genes were found that were both significantly associated with climatic conditions and also identified in selective sweeps, of which 28, 95, 22, and 85 genes were detected in the western, central, southern, and eastern groups, respectively (Supplementary Table S6). A subset of these genes may underlie the mechanism of adaptation to diverse abiotic factors in *F. nilgerrensis*.

The enrichment analysis was performed to identify previously characterized genes or biological pathways known to be involved in adaptation to distinct environments. For the western group, we found that genes related to response to stimulus, salt stress, and osmotic stress were highly overrepresented. Specifically, the Ultraviolet-B receptor UVR8 (*UVR8*; evm.model.ctg32.2005) gene and the Aspartic proteinase A1 (*APA1*; evm.model.ctg28.472) gene were identified to be under strong selection. The *UVR8* gene in *Arabidopsis* is involved in plant acclimation and thus promotes survival in sunlight [39], and the *APA1* gene is known to be involved in drought tolerance in *Arabidopsis* [40]. These might be linked to adaptation to the high-altitude environment in the Hengduan Mountains. For the central group, we found genes implicated in the regulation of flowering time (*HAC1*) [41, 42], blue light responses (*CRY1* and *CRY2*) [43], toxic heavy metal ion responses (*CNGC1* and *CNGC10*) [44], response to changes in humidity (*SAGL1*) [45], and so on. For the southern group, we found genes related to plant development (*At5g45160*) [46], fruit development (*GRDP1*) [47], and chemical-induced genotoxic and oxidative stress (*NPC1*) [48]. For the eastern group, we discovered several genes involved in regulating plant growth (*CGR2*, *ERG28*, *TOR*) [49–51].

## Discussion

The population genomic approach provides a novel perspective for deciphering patterns of genetic variation and structure, and demographic history. Furthermore, recent progress in genomic tools enables the identification of the adaptive genomic footprints shaped by heterogeneous environments, contributing to understanding how climate has shaped and will continue to shape the genome of this species. In this study, we sequenced 193 individuals from 28 geographical populations to obtain genome-wide SNPs of *F. nilgerrensis*. Based on genetic structure analysis, we found that *F. nilgerrensis* was roughly divided into two main clades using the Δ*K* method, which separated the populations in the Hengduan Mountains from other populations. However, a previous study pointed out that the Δ*K* method frequently identifies *K* = 2 as the top level of hierarchical structure, even when more subpopulations are present [38]. Both our PCA and phylogenetic analysis indicated fine-scale structure within *F. nilgerrensis*. Thus we considered four groups according to their genetic composition and geographic distribution. We found that the western group located in the Hengduan Mountains exhibits the highest level of genetic diversity (Table 1), followed by the central group, which is located to the east of the Hengduan Mountains. This has been observed previously in other plant species, such as *Quercus aquifolioides* [52], *Taxus wallichiana* [53], and *Circaeaster agrestis* [54]. The western group also had the greatest Tajima’s *D* value, indicating that intermediate-frequency alleles appeared more frequently than other groups [55]. Our results suggested that the Hengduan Mountains were the center of genomic diversity of *F*. *nilgerrensis*. This result supports the hypothesis that the Qinghai-Tibet Plateau and the adjacent area were the glacial refuges of *Fragaria*, which could explain why southwest China was the center of species diversity for this genus [56].

Environmental factors have been widely reported to drive differential selective pressures leading to genetic divergence during adaptation to heterogeneous environments [57, 58]. We identified a high level of genetic differentiation among *F. nilgerrensis* groups. The analyses of IBD and IBE suggest that both geographic and environmental factors have contributed to the genetic differentiation observed within this species. The geographic distance explained 11.58% of the observed variance, and a strong pattern of isolation by distance (0.5039) was observed between populations, suggesting the considerable contribution of geographic isolation to genetic variation. The complex topography and natural barriers such as the north–south mountain series of the Hengduan Mountains might limit the dispersal between the three genetic groups in the southwest to a certain extent. However, the substantial collinearity observed between geographic and environmental distances made it difficult to disentangle the relative contributions of geographic and environmental factors in shaping the genomic variation. Thus, we further performed RDA to quantify the relationship of genomic variation with climate and geography. RDA analysis identified that both climatic factors and geographic distance shaped a significant proportion of genomic variation: 12.25 and 11.58% respectively. Despite the large proportion of collinearity (28.29%) between environment and space, our analysis identified that specific environmental factors such as temperature seasonality and precipitation seasonality explained a substantial portion of SNP variation among populations when the effects of spatial structure were also considered. Our study supplements and reinforces some previous findings [59, 60] in showing that temperature and precipitation might be important factors driving ecological adaptation in *Fragaria* species. Johnson *et al*. [59] found that the most easily altered niches of *Fragaria* were the coefficient of variation of precipitation seasonality, annual mean temperature, temperature seasonality, and mean altitude. Similarly, Yang *et al*. [60] found that altitude, temperature, and precipitation were the dominant environmental variables that affect the potential spatiotemporal dynamics patterns of six wild strawberry species, including *F. nilgerrensis*.

We investigated the long-term changes in effective population size of the four different groups and uncovered evidence for changes in effective population sizes post-Pleistocene. The geological epoch following the most recent glaciation event (LGM) is associated with dynamic shifts in climates worldwide. During and/or following the LGM, four groups experienced population declines followed by subsequent population expansions. These patterns highlight that *F. nilgerrensis*, like most plant species, is sensitive to temperature changes. Our findings revealed that temperature seasonality was the strongest climate predictor of the degree of genetic differentiation among the four groups. Interestingly, the southern group located in Yunnan slightly expanded during the LGM. This scenario had been previously reported for several other organisms, such as *Primula obconica* [61] and *Microvelia douglasi* [62].

Identifying the genomic regions that evolved in response to various abiotic factors in strawberry species could contribute to furthering our understanding of the ability of populations to sustain or respond to rapid changes in the environment [35, 63, 64]. The PCA of 19 climatic variables suggested that the four genetic groups we identified within *F. nilgerrensis* were, in general, significantly diverged from each other based on their native environments (Supplementary Fig. S5c). Despite the relatively large number of SNPs associated with environmental variables, it is difficult to test whether these SNPs are explained by selection [35]. Thus, we retained the genes identified by the GEA analysis that overlapped with the selective region to reduce the potential false-positive rate and identify loci that encode adaptations in response to changes in the environment with higher confidence. We detected only a few shared genes associated with climatic variables between any two groups, which further supports group-specific adaptation to different climates in *F. nilgerrensis* on the genomic level. For example, the western group located in the Hengduan Mountains has an average elevation of 2902 m and is exposed to lower temperatures, reduced levels of oxygen, and higher ultraviolet radiation compared with the rest of the range of the species. The gene *UVR8* we detected in the western group encodes an ultraviolet-B (UV-B) light receptor previously shown to be involved in UV-B sensing and tolerance in other species [65]. Plants recognize exposure to UV-B using this photoreceptor and activate downstream signal transduction pathways to initiate acclimation to UV-B rays [65]. The gene *HAC1* detected in the central group played an important role in vegetative and reproductive development; a previous study suggested that it is essential for regulating flowering time, and lesions in *HAC1* can cause a late-flowering phenotype in *Arabidopsis* [41]. The central group is located to the east of the Hengduan Mountains, a transitional zone between high- and low-altitude areas. Its special environment may cause some traits to be selected. In the other two groups, with relatively low altitudes, we found some genes related to vegetative growth, e.g. the *At5g45160* gene found in the southern group and the *CGR2*, *ERG28*, and *TOR* genes found in the eastern group. Selection on genes involved in vegetative growth has previously been reported in populations from relatively low altitudes in *Arabidopsis lyrata* [66]. Previous studies on *Arabidopsis thaliana* [67] also showed that low-altitude populations have higher leaf count and larger siliques than high- and middle-altitude populations. Overall, our analyses in *F*. *nilgerrensis* provide new information about the loci related to the adaptive responses to diverse abiotic stresses, and provide prime candidates for future functional research and potential molecular markers to guide breeding efforts in strawberry.

In summary, we report new genomic resources for *F*. *nilgerrensis* and provide novel insights into the population structure and demographic history of this CWR. We explicitly measured the relative impact of geographic and environmental variables on population divergence to dissect these two features in shaping patterns of observed genomic variation. Our analysis identified that climatic variables explained more genomic variation than geographic distance, with temperature seasonality explaining the most SNP variation when conditioned on spatial structure, which suggests that local adaptation greatly promotes population genetic differentiation. By combining selective sweep analysis and GEA, we identified several candidate genes possibly related to adaptation to heterogeneous climate environments. Our results provide many avenues for conservation and utilization of *F*. *nilgerrensis* germplasm and the breeding of cultivated strawberries that can grow in environments affected by climate change.

## Materials and methods

### Sample collection, DNA extraction, and sequencing

We collected 193 samples from 28 populations (3–10 specimens per population) across the distribution range of *F. nilgerrensis* in China (Supplementary Table S1). We extracted the genomic DNA from leaves using the DNA Plant Kit (AU31111-16, Bioteke, Beijing). DNA libraries were prepared for each sample and sequenced by Novogene Bioinformatics Institute (Beijing, China) using the Illumina Novaseq 6000 platform (San Diego, CA) with paired-end 150-bp reads.

### Data processing, mapping, and variant calling

For raw sequencing reads, we (i) removed reads with >10 nucleotides aligned to the adapter, allowing ≤10% mismatches, and (ii) filtered out low-quality read pairs including reads with >10% unidentified nucleotides (N) and with >50% of base quality ≤5 in either of the paired reads. All clean reads were then mapped to the reference genome of *F. nilgerrensis* [37] (272 Mb) using the BWA-MEM aligner with default parameters using bwa-0.7.17 [68]. The resulting bam files were sorted using SAMtools [69], and duplicated reads due to DNA amplification by PCR were removed using the Picard v2.20.2 MarkDuplicates tool (http://broadinstitute.github.io/picard/). After that, SNP calling in each individual was performed using HaplotypeCaller of GATK v4.1.4.0 [70] to generate intermediate genome Variant Call Formats (gVCFs), and then all individuals were jointly genotyped using GATK GenotypeGVCFs. Only sites with base quality ≥30 were used in HaplotypeCaller. To minimize the influence of mapping bias, variant sites were filtered using GATK VariantFiltration with filter expression QD < 2.0 || FS > 60.0 || MQ < 40.0 || MQRankSum < −12.5 || ReadPosRankSum < −8.0. Sites showing an extremely low (<8×) or high (>200×) average coverage were also filtered out. Finally, a total of 9 231 119 sites with missing rate <20% were left for further analysis, ~33 938 SNPs per megabase. Remaining filtration was done according to the requirement of each analysis performed below.

### Population structure and genetic diversity

We used a Bayesian clustering approach implemented in the software STRUCTURE [71] to delineate the cluster of each sample. We ran 10 independent runs for each *K* value from 2 to 10, where the length of the burn-in period and number of MCMC replications after burn-in were set to 50 000 and 100 000, respectively. We used STRUCTURE HARVESTER [72] to detect the most probable number of *K* groups through the Evanno method [73]. The cluster assignment across replicate runs was averaged using CLUMPP [74] and the output was plotted using DISTRUCT [75]. We also used PCA implemented in GCTA [76] to assess population structure. In addition, we constructed a neighbor-joining tree based on the *p*-distance model using MEGA X [77] with 1000 bootstrap replicates. For these analyses, we filtered out sites with minor allele frequency <5% and performed a linkage disequilibrium (LD)-based SNP pruning process in PLINK v1.90 (option —indep-pairwise 50 5 0.2) to exclude strong linked SNPs. Specifically, this procedure calculates LD (*r*^2^) between each pair of SNPs within a sliding window of 50 SNPs with a step of 5 SNPs and removes one of a pair of SNPs if *r*^2^ > .2.

After clarifying the population structure of *F. nilgerrensis* based on genetic clustering and phylogenetic analysis, we used VCFtools [78] to calculate population genetic statistics including nucleotide diversity (*π*), Tajima’s *D* for each group, and population- and group-level pairwise *F*_ST_. Specifically, we computed *π* per site using the parameter -site—pi on all SNPs of individuals from each group. The total nucleotide diversity for each group was computed by summing the *π* values of all SNPs and dividing by the total number of callable sites. Tajima’s *D* and pairwise *F*_ST_ were calculated in a non-overlapping 20-kb sliding window.

### Population demography

We used MSMC v2 [79] to reconstruct the history of changes in *N*_e_ through time. Prior to performing the analysis, all segregating sites within each group were phased and imputed using Beagle v4.0 [80]. We then inferred the historical changes in *N*_e_ of the four genetic groups based on sets of two individuals (four haplotypes), respectively. For each group, 50 rounds of random samplings were run to determine the mean and standard deviation of *N*_e_ changes. The input files for MSMC analysis were generated according to MSMC Tools (https://github.com/stschiff/msmc-tools). One-year generation times and a mutation rate of 7 × 10^−9^ substitutions per site per year were used to estimate times and population sizes.

We also used the sequential Markovian approach implemented in SMC++ [81] to infer the historical changes of *N*_e_. SMC++ takes advantage of both information contained in the site frequency spectrum and LD to make demographic inferences. In addition, SMC++ is phase-insensitive, limiting switch errors in phasing that can bias *N*_e_ estimates for recent times. A polarization error of 0.5 was used since the identity of the ancestral allele could not be determined for many loci. The years for a generation and the mutation rate were set as MSMC.

### Genetic, geographic, and environmental correlations

To illustrate the effects of geographic and environmental variables on shaping genetic structure, we conducted IBD and IBE analyses to assess associations between pairwise *F*_ST_ and geographic distance and environmental distance by the Mantel test with 10 000 permutations implemented in the R package vegan v2.5.4 [82]. We calculated pairwise geographic distances among 28 populations using the distHaversine function in the R package geosphere v1.5–10 [83]. The 19 bioclimatic variables downloaded from WorldClim 2 [84] were used at 30 arc-seconds resolution (Supplementary Tables S7 and S8). We first performed a PCA on these climatic variables using JMP 13.0.0 (SAS, Cary, NC), then used the first two principal components as points in two dimensions to calculate a pairwise environmental distance matrix for all populations.

We further performed RDA to estimate the degree to which genome-wide SNP variation among populations is explained by geographic or environmental variables. To avoid the influence of multicollinearity, we eliminated one of the variables in each pair with a correlation value >0.9 through Pearson correlation analysis and retained the remaining nine variables. These nine climate variables were further tested using the forward.sel function in the R package adespatial [85] to identify predictive and non-redundant environmental variables for variance partitioning. Prior to running the RDA, we estimated the spatial genetic structure from geographic coordinates based on dbMEMs [86]. dbMEMs are orthogonal spatially explicit eigenvectors that are able to model any type of spatial structure, including broad-, medium-, and fine-scale patterns [87]. We used the dbMEM function in the adespatial package to calculate dbMEMs. Forward selection was implemented using the forward.sel function in the adespatial package to reduce the number of variables in the model, with a significance level for each tested variable set at 0.01 and a maximum limit for adjR2thresh equal to the adjusted *R*^2^ of the RDA model including all initial variables. The RDA analysis was performed using the rda function in vegan. The overall significance and the significance of each variable were assessed using the anova.cca function in vegan with 999 permutations.

### Detection of selective sweeps

We performed scans on each group separately by using two approaches: (i) a method relying on multiple signatures of a selective sweep via the enumeration of SNP vectors (Raised Accuracy in Sweep Detection, RAiSD) [88] and (ii) a haplotype-based statistics method (XP-nSL) [89], which was implemented in Selscan v1.3.0 [90]. RAiSD collectively utilizes three distinct signatures to detect selective sweeps: local reduction of the polymorphism, the shift in the site frequency spectrum toward low- and high-frequency-derived variants, and the localized pattern of LD [88]. RAiSD calculates the *μ* statistic across the genome from SNP-driven, overlapping windows. We calculated *μ* using default settings in four groups separately. Finally, the *μ* statistics were averaged across non-overlapping 20-kb windows on each chromosome. Windows with <10% of covered sites left from previous quality-filtering steps were excluded. Only the top 10% of windows for each group were retained for downstream analysis.

XP-nSL summarizes haplotype diversity by calculating the average number of variant sites in a genomic region that are identical across all haplotypes, and then compares haplotype pools between two different populations, which makes it possible to detect differential local adaptation [89]. XP-nSL was calculated on all nine comparisons of the four groups (for each comparison, using each group once as the objective and once as the reference). We first calculated the raw XP-nSL scores with the default parameters, then normalized them across non-overlapping 20-kb windows based on the genome-wide empirical background using norm v1.3.0 (https://www.github.com/szpiech/selscan). Using either of the other groups as the reference, for each group, windows' with the highest fraction of extreme scores higher than the 99th percentile of its distribution were identified as candidate regions.

The overlaps of the results from the two methods were identified and regarded as the high-confidence selective sweep regions. We then identified genes that localized within or were closer than 5000 bp to the selective sweep regions to exclude the border effect [91].

### Genome–environment association analysis

To detect genome-wide signatures of local adaptation, we applied two GEA tests that can infer SNPs that have significant associations with specific environmental factors. First, we tested for the correlation of environmental covariates with SNPs using the standard covariate model in BayPass [92]. For a comprehensive consideration of the environmental effect, the first two environmental principal components, which explained 84.7% of the total variance (Supplementary Fig. S5b and c), were kept to represent the environmental covariates for further analysis. The output (population covariance matrix) from this method was directly estimated with the core model. To do this, we generated a set of 10 000 putatively neutral and independent SNPs by thinning the intergenic and 4-fold degenerate sites, and then used them to estimate the population covariance matrix Ω, which indicates the degree of relatedness between populations. We repeated this step three times based on different SNP subsets generated randomly, and each subset was run with three different seeds. We used paired Forstner and Moonen Distance (FMD) [94] to compare the resulting covariance matrices in pairs. The paired FMD between different seeds in the same subset are between 0.95 and 1.26, and the paired FMD distances between different subsets are between 1.52 and 2.02. We took the average of the results of the nine matrices to get the final covariance matrix Ω. By introducing the population covariance matrix Ω estimated with the core model and the correlation coefficients, which had a uniform prior distribution between −.3 and .3, we ran the standard covariate model five times with different seeds. Finally, we used the median computed over five different independent runs as an estimate. According to Gautier [92], covariates with an empirical Bayesian *P*-value (eBPmc) >3 were considered significantly associated.

We then performed RDA, a multivariate constrained ordination method, to identify SNPs associated with environment factors. RDA has been found to show a better trade-off between false-positive and true-positive rates across weak, moderate, and strong multilocus selection, and can detect processes that result in weak, multilocus molecular signatures [95]. Partial RDA enables the use of geographic location to condition all linear regressions to spatial structure. We used the anova.cca function and 999 permutations in vegan to assess significance for the full model and each constrained axis to be evaluated for candidate loci. The constraint axes with *P*-value <.05 were considered significant and were used to evaluate candidate SNPs. Candidate SNP for the first four RDA axes were identified as being +/− three standard deviations from the average RDA loading, creating a cutoff of two-tailed *P-value* of .0027 (Supplementary Figs S8 and S9).

### GO term enrichment

To determine if any functional classes of candidate genes were over-represented, we performed GO analysis using the R package topGO v2.38.1 [96]. Fisher’s exact test was used to calculate the statistical significance of enrichment, and GO terms with *P*-values <.05 were considered as interesting biological processes. As described in the topGO manual, the False Discovery Rate/Family-Wise Error Rate adjustment process can produce a very conservative *P*-value, resulting in some interesting GO terms being lost.

## Acknowledgements

This work was supported by the Strategic Priority Research Program of the Chinese Academy of Sciences (XDB31000000) and the National Natural Science Foundation of China (31501799) to M.K., and the National Science Foundation (2029959) and United States Department of Agriculture (2020-67013-30870) to P.E.

## Author contributions

M.K. conceived and designed the project. Y.H., C.F., and L.Y. performed the sampling and experiments, and the data analysis. Y.H. and M.K. wrote the manuscript. All authors read and approved the final manuscript.

## Data availability

The whole-genome sequencing (WGS) raw reads have been deposited in the National Center for Biotechnology Information Sequence Read Archive (SRA) database under BioProject accession number PRJNA748880.

## Conflict of interest

The authors declare no competing interests.

## Supplementary data

Supplementary data is available at *Horticulture Research* online.

## Supplementary Material

Web_Material_uhab059
