# Supplementary material for: Genomic population structure and local adaptation of the wild strawberry Fragaria nilgerrensis
Source: Hortic Res. 2022 Jan 19;9:uhab059. doi: 10.1093/hr/uhab059 (PMC8993681; doi:10.1093/hr/uhab059)
Supplement: Web_Material_uhab059 [file web_material_uhab059.zip › 5918_1_supp_0_r1r4m0.docx]

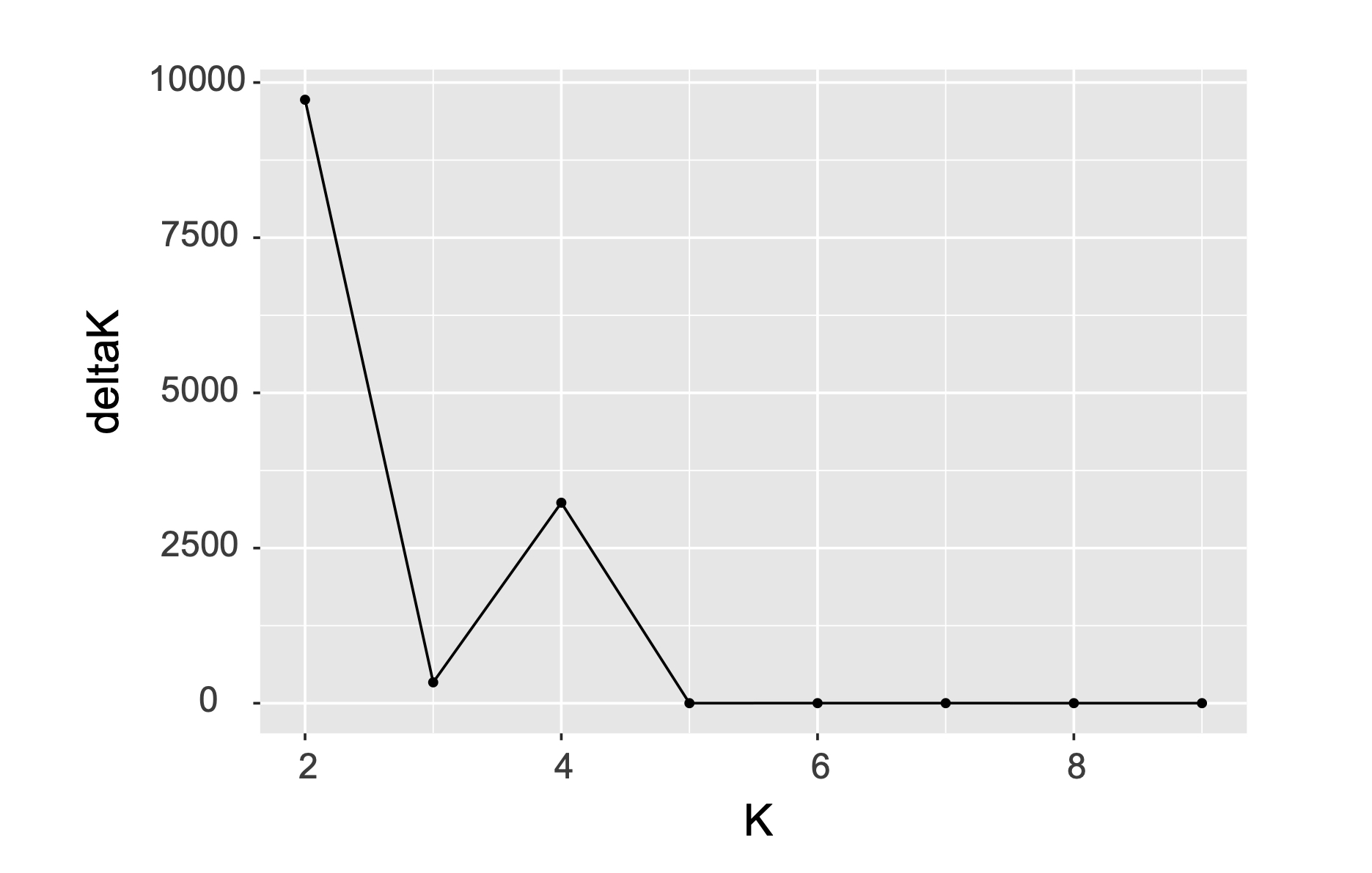


**Figure S1** ∆*K* estimated from log likelihood in STRUCTURE for different *K* values.


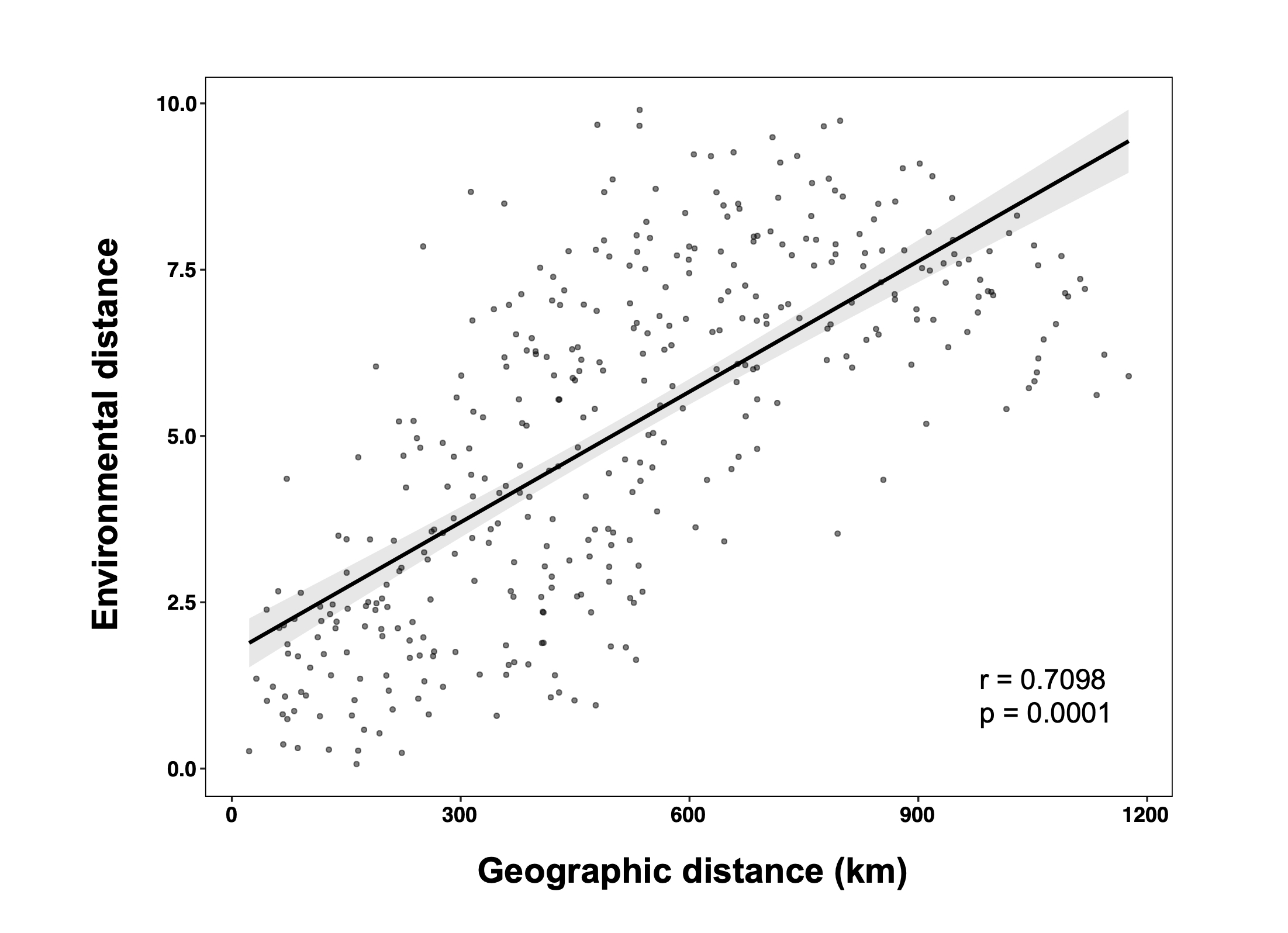


**Figure S2** Environmental distance plotted against geographical distances between populations.


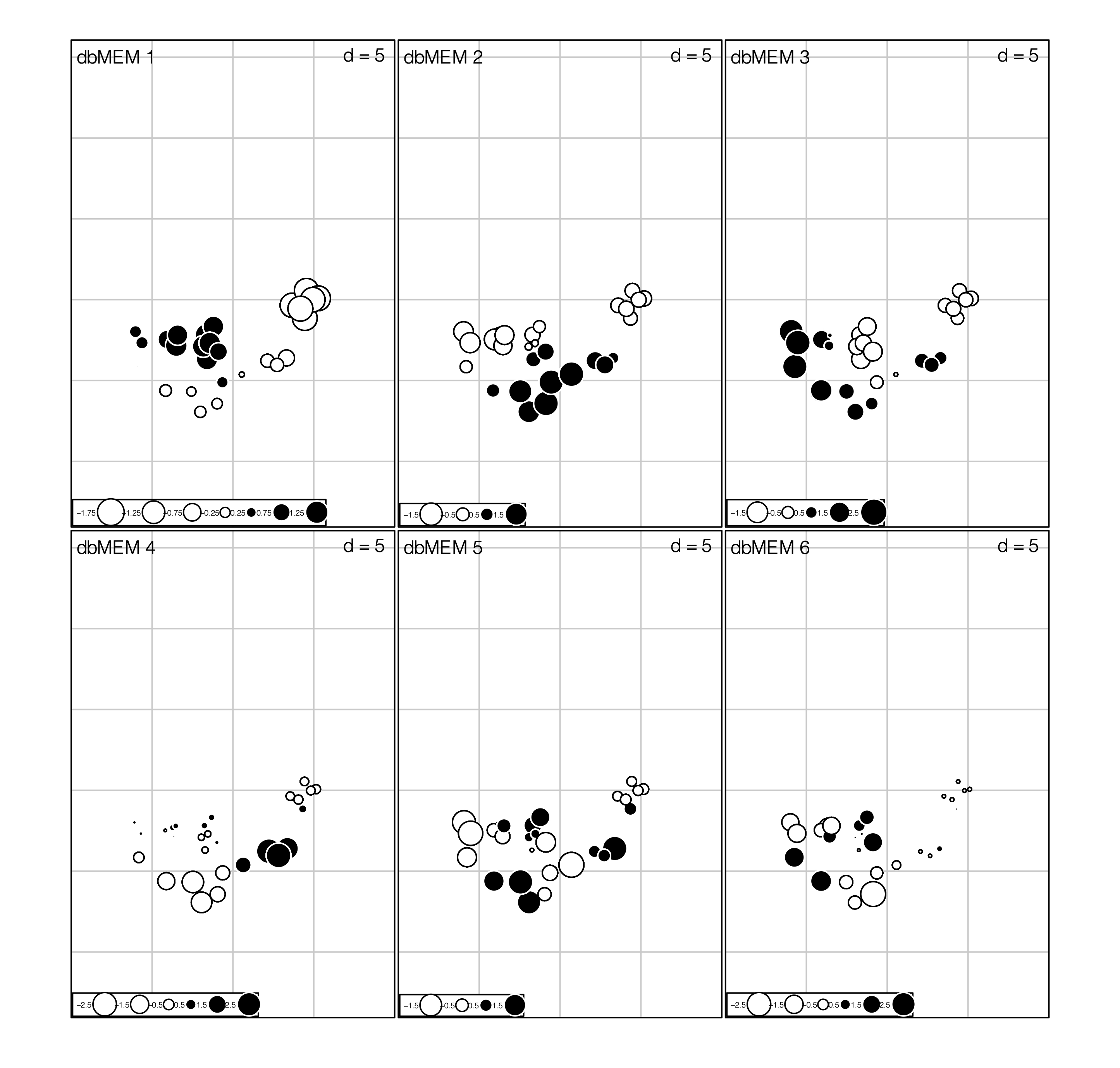


**Figure S3** Bubble plots of the 6 significant dbMEM variables. The color and size of the points correspond to the sign (+ or -) and magnitude of the dbMEM variables, respectively.


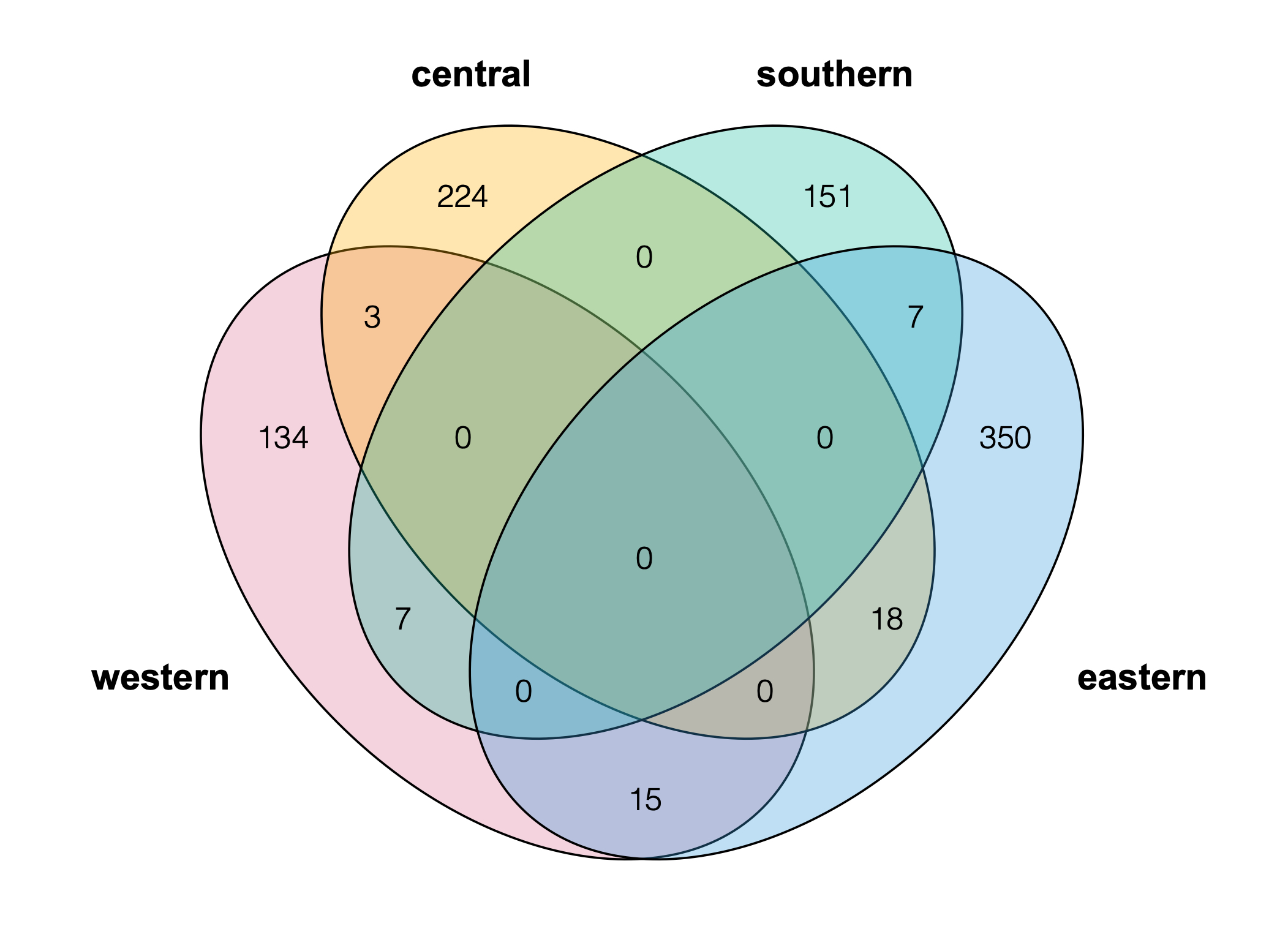


**Figure S4** Venn diagram showing the overlap of candidate genes within selective sweep regions of four groups.


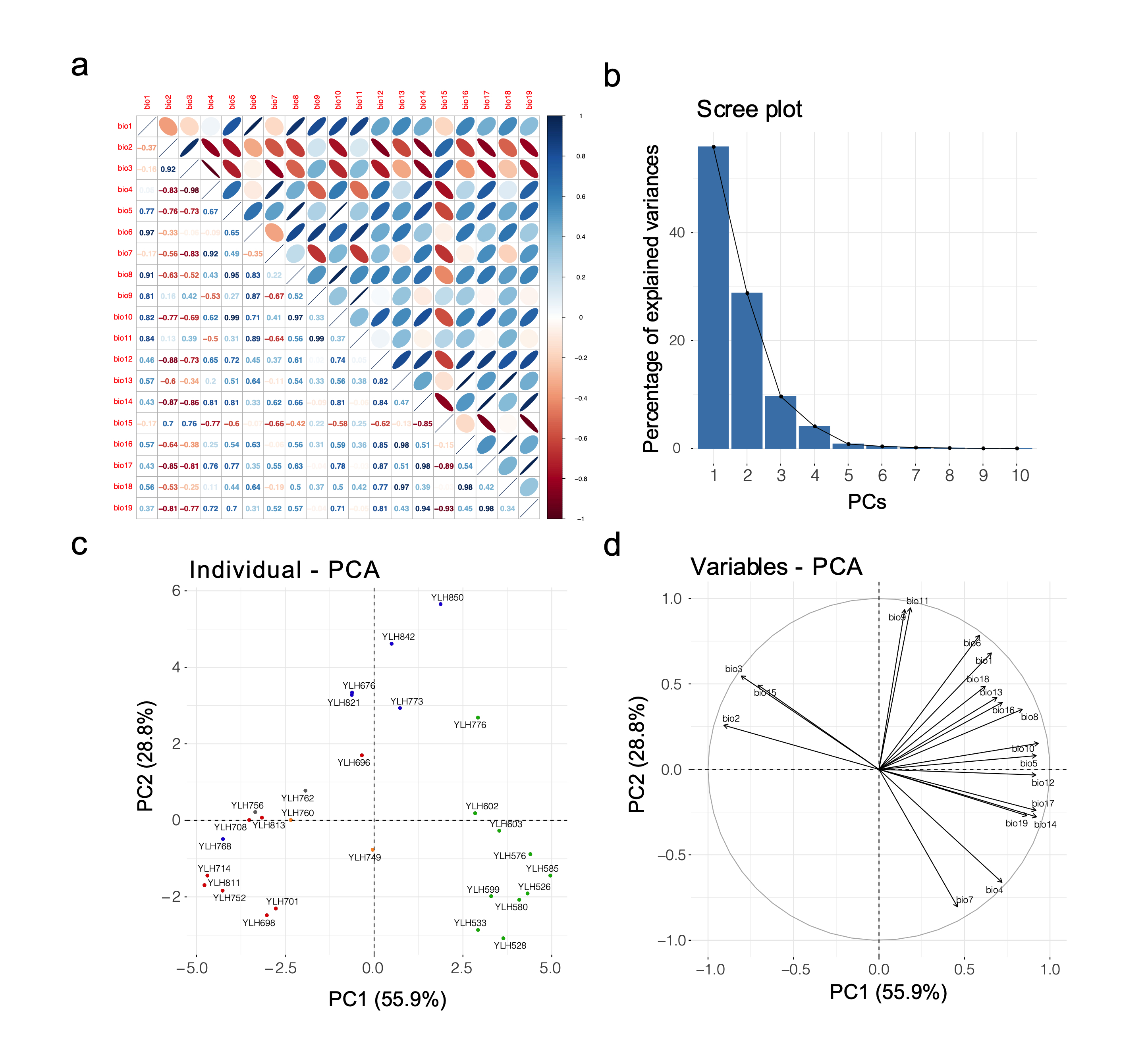


**Figure S5** (a) Correlation between 19 climatic variables. Positive correlations are displayed in blue and negative correlations in red color. Color intensity and the size of the circle are proportion to Pearson’s correlation coefficient. (b) Eigenvalues contribution to the variance in principal component analysis for all 19 climatic variables. (c) All populations loaded on the top two PCs. Colors correspond to four genetic groups shown in Figure 1. (d) 19 environmental variables loaded on the top two PCs.


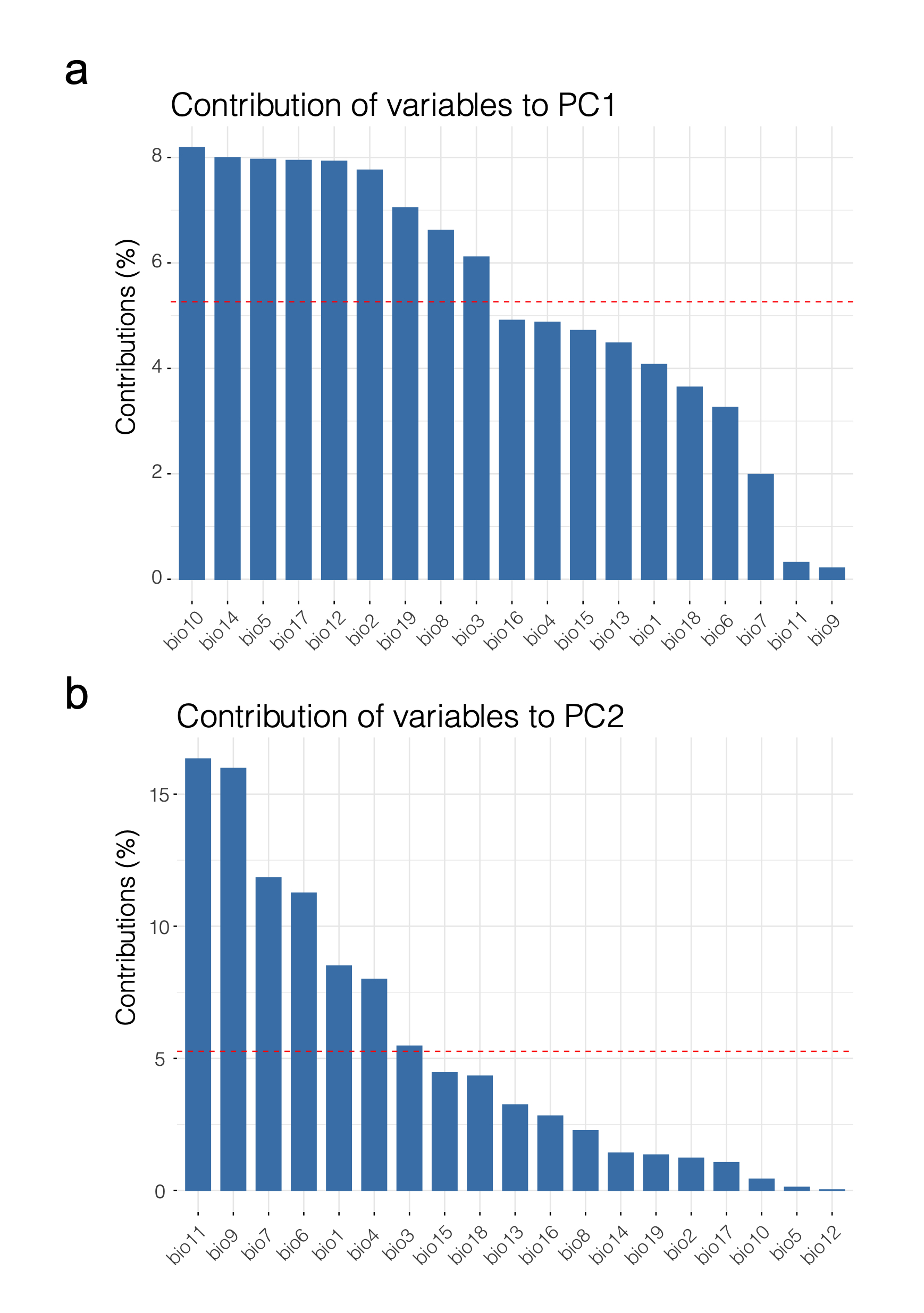


**Figure S6** (a) The proportion of contribution for each climatic variable along PC1. (b) The proportion of contribution for each climatic variable along PC2. The red line indicates the threshold of equal contribution across all variables.


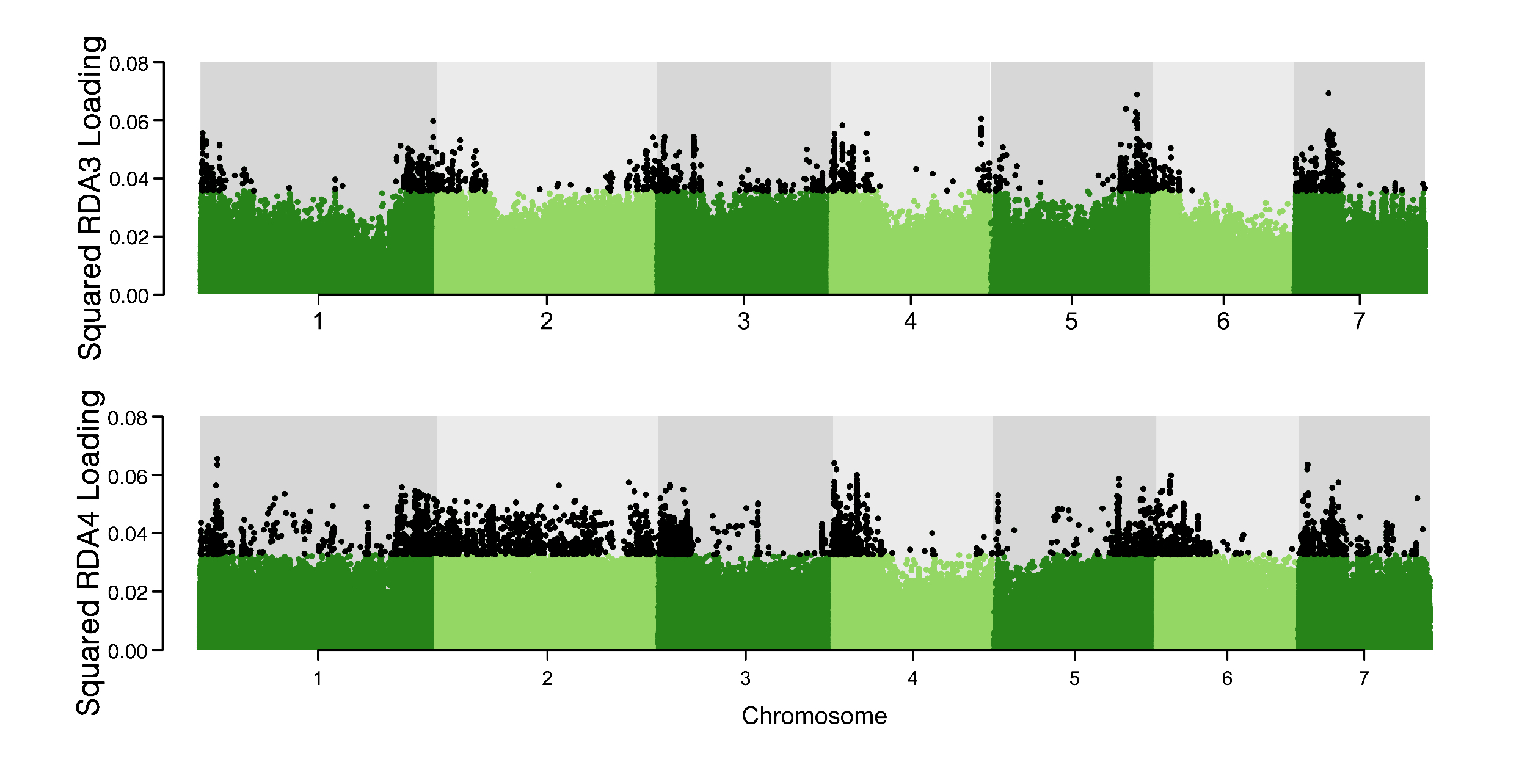


**Figure S7** A Manhattan plot for the third RDA axis (top panel) and the fourth RDA axis (bottom panel) for the climate variables. The black dots represent SNPs with significant associations along the RDA axes (at least three standard deviations away from the mean squared loadings).


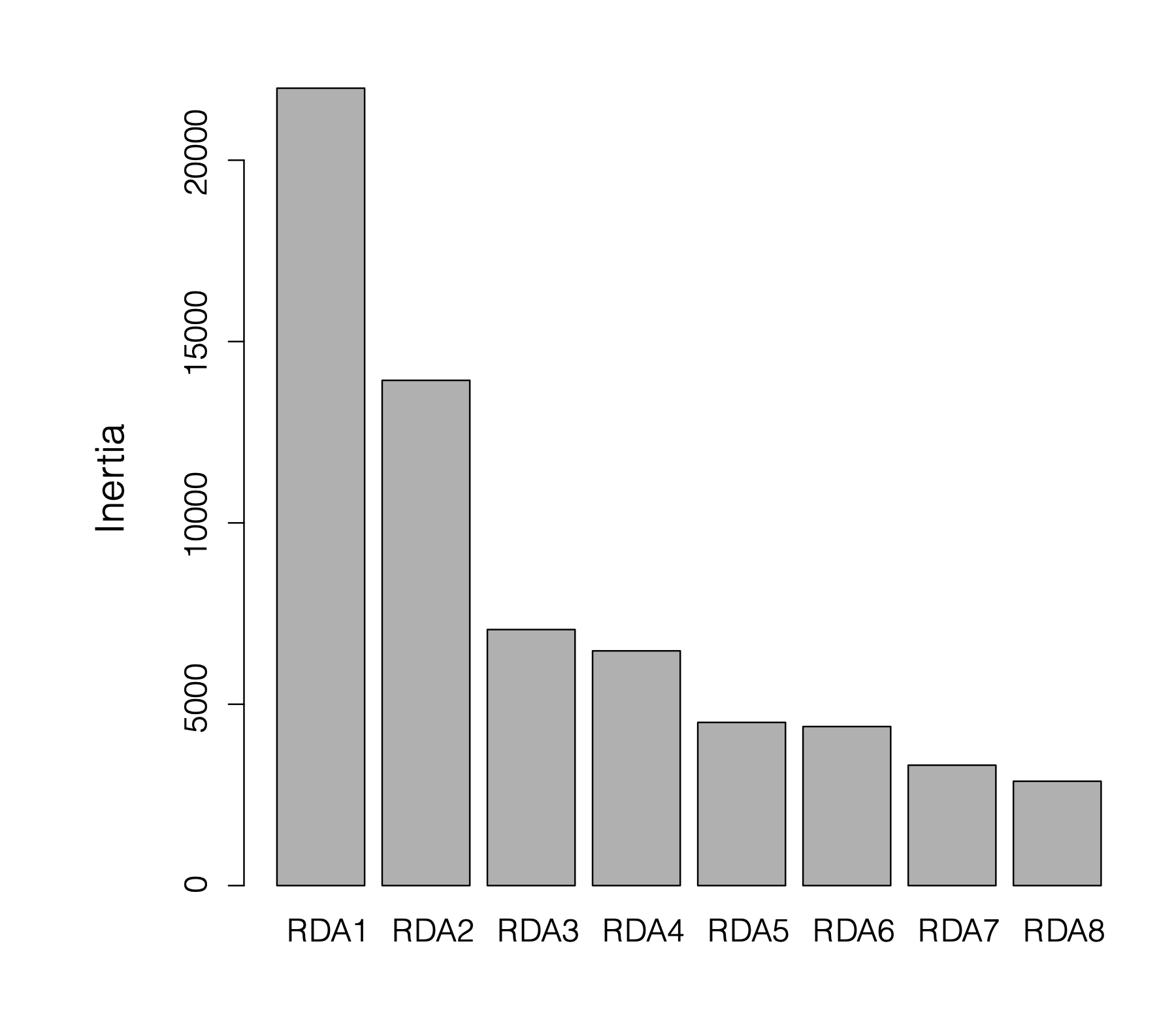


**Figure S8** Partial RDA scree plot. Amount of variance (inertia) accounted for by each of the RDA axes.


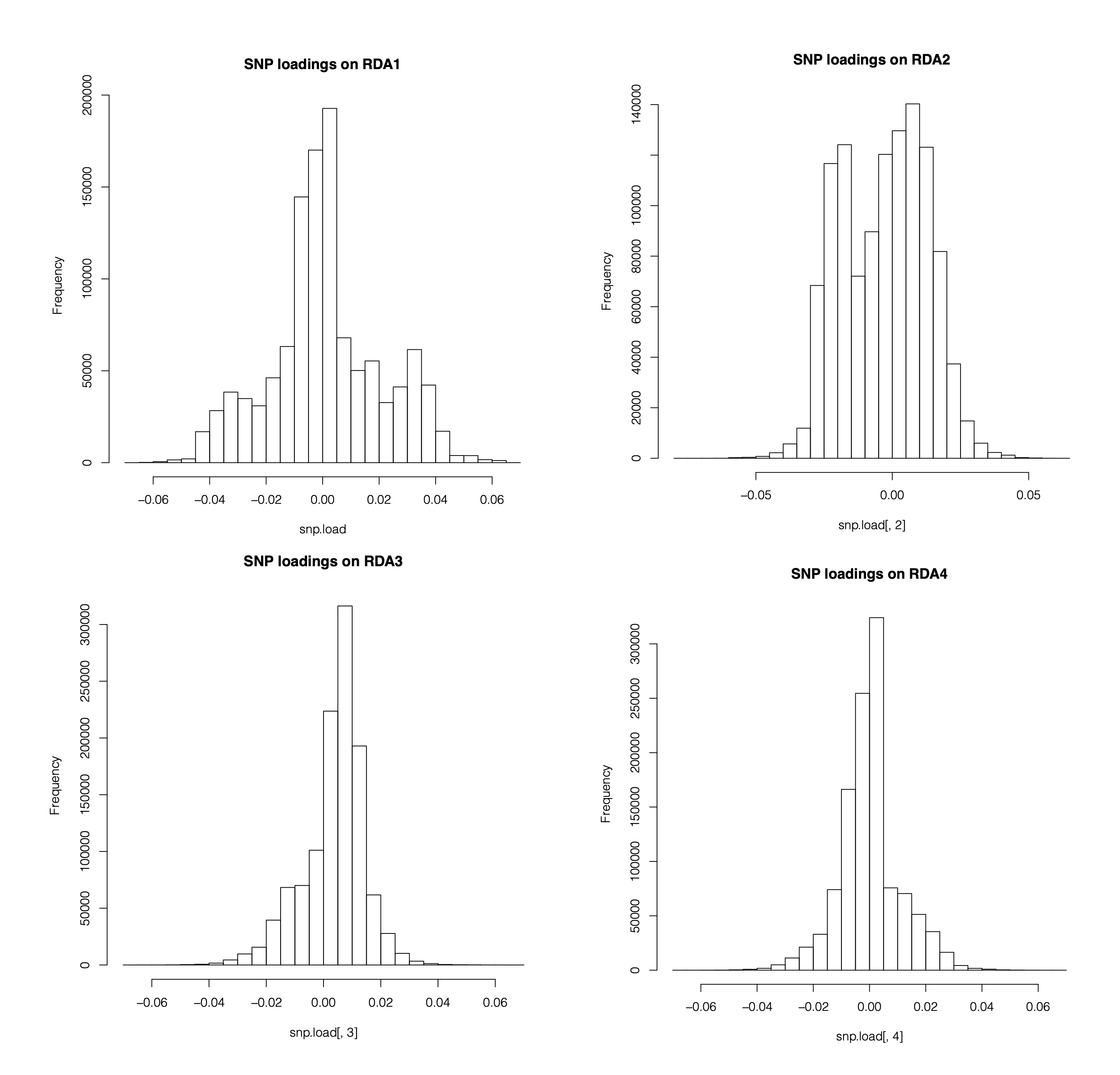


**Figure S9** The SNP loadings on the first four RDA axes.
